# iGMDR: Integrated Pharmacogenetic Resource Guide to Cancer Therapy and Research

**DOI:** 10.1016/j.gpb.2019.11.011

**Published:** 2020-09-08

**Authors:** Xiang Chen, Yi Guo, Xin Chen

**Affiliations:** 1 Institute of Pharmaceutical Biotechnology and the First Affiliated Hospital Department of Radiation Oncology, Zhejiang University School of Medicine, Hangzhou 310058, China; 2 Department of Polymer Science and Engineering and Key Laboratory of Adsorption and Separation Materials and Technologies of Zhejiang Province, Zhejiang University, Hangzhou 310027, China; 3 Joint Institute for Genetics and Genome Medicine between Zhejiang University and University of Toronto, Zhejiang University, Hangzhou 310058, China

**Keywords:** Genetic model, Pharmacogenetics, Anticancer drug, Cancer, Drug response

## Abstract

Current pharmacogenetic studies have obtained many genetic models that can predict the therapeutic efficacy of anticancer drugs. Although some of these models are of crucial importance and have been used in clinical practice, these very valuable models have not been well adopted into **cancer** research to promote the development of cancer therapies due to the lack of integration and standards for the existing data of the pharmacogenetic studies. For this purpose, we built a resource investigating **genetic model** of **drug response** (iGMDR), which integrates the models from *in vitro* and *in vivo* pharmacogenetic studies with different omics data from a variety of technical systems. In this study, we introduced a standardized process for all integrations, and described how users can utilize these models to gain insights into cancer. iGMDR is freely accessible at https://igmdr.modellab.cn.

## Introduction

After the completion of the Human Genome Project, pharmacogenetics has been presented as a promising field and has been extensively studied [1], [2]. Pharmacogenetics integrates pharmacology and genetics as a single discipline to correlate the genetic characteristics and drug responses of an organism. The genetic characteristics include not only those at the genome level but also those at any level of omics related to gene function, such as the transcriptome and proteome [3]. The goal of pharmacogenetics research is to find more efficient strategies for disease therapies based on personalized genetic characteristics, which is one of the major bottlenecks in implementing personalized medicine at the current stage [4]. The selection of therapeutic strategies of a disease is almost always based on genetic knowledge of a population and often fails for specific individual cases [5], [6]. With the accumulation and development of the sequencing technologies and pharmaceutical research methods, the relationship between the curative effects of some drugs and the genetic characteristics of individuals has become increasingly visible, thus leading to more accurate prediction of the effect of therapeutic strategies based on the genetic characteristics of patients and producing genetic models with extensive clinical application [7].

Cancer development has been considered to be closely related to genetic dysfunction, and understanding this dysfunction is therefore the main goal of precision medicine and personalized medicine [5]. To this end, pharmacogenetic studies in cancer have been widely implemented, producing various genetic models that have been shown to be effective in clinical practice [8], [9]. Nevertheless, the pace of these studies has lagged far behind what is required by precision medicine for cancer, and a large proportion of cases received therapies from traditional “one treatment fits all” strategies. More systematic studies and more effective data analysis are critical to obtain enhanced precision models. Currently, there are several *in vitro* studies on the pharmacogenetics of cancer cell lines, such as the Cancer Cell Line Encyclopedia (CCLE, https://portals.broadinstitute.org/ccle) [10], Genomics of Drug Sensitivity in Cancer (GDSC, https://www.cancerrxgene.org/) [11], Cancer Therapeutics Response Portal (CTRP V2, http://portals.broadinstitute.org/ctrp/?page=#ctd2BodyHome) [12], and MD Anderson Cell Lines Project (MCLP, http://tcpaportal.org/mclp/) [13]. Experimental design from *in vitro* to *in vivo* is one of the main approaches for acquiring knowledge that will be applied to clinical practice. Fortunately, these studies have yielded many genetic models for both specific anticancer drugs and specific cancers. In addition, *in vivo* studies of model organisms and xenograft models have yielded considerable preclinical genetic models for cancers. Some of these models have been approved by the Food and Drug Administration (FDA, https://www.fda.gov) of the United States as the guidelines for therapies compiled into the National Comprehensive Cancer Network (NCCN,https://www.nccn.org/). However, the results of these studies still exist in isolation, without effective integration and utilization. While there are still many trials and challenges ahead before these studies yield genetic models for clinical use, the value of these data should not be underestimated. The goal of building the resource investigating genetic model of drug response (iGMDR) is to collect these models from different pharmacogenetic studies involving various technical approaches and sources in order to obtain new insights into cancers.

**Figure 1:**
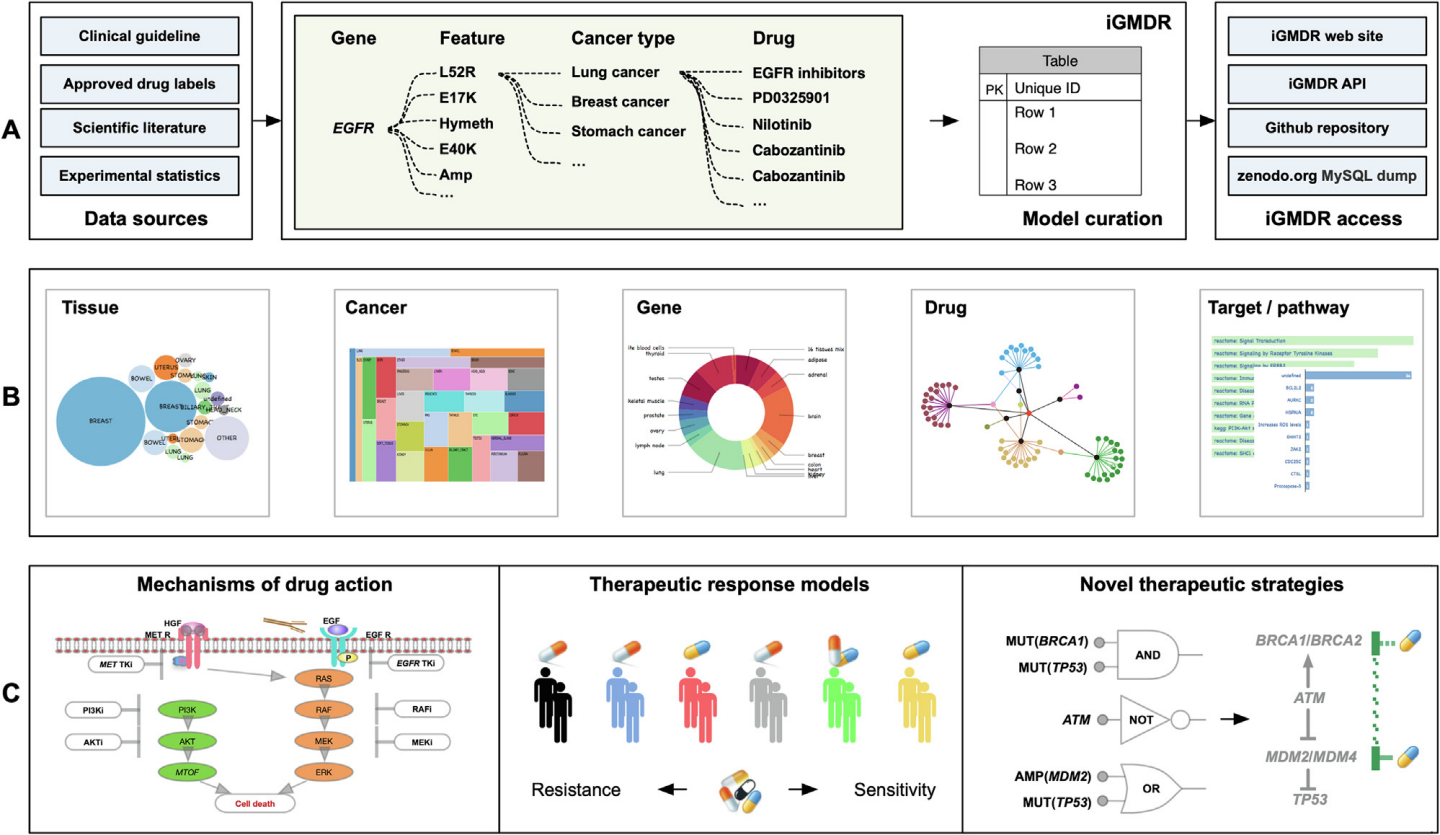
**Flowchart of pharmacogenetics model data integration and application** **A.** Processes of data collection and collation in iGMDR. **B.** User interface of the genetic model data and the data visualization display. **C.** Case demonstration of the application of the pharmacogenetic models. Applications include designing a new panel based on the model combination to discover the mechanism of drug action, using the model for personalized anticancer therapy, using information from models, relevant pathways, and targets to design more effective therapeutic strategies.

In this work, we collected *in vitro* and *in vivo* models including clinical practices from authoritative clinical institutions, preclinical studies from the literature review, as well as drug sensitivity tests from cancer cell lines, and obtained over 154,000 models (Figure 1A). The models were obtained by using technical systems from the perspectives of the genome, epigenome, transcriptome, and proteome. Whole genome/exome sequencing (WGS/WES) are used for genome studies, to obtain information on copy number variation (CNV), single nucleotide variation (SNV), and structural variation (SV). Whole-genome bisulfite sequencing and methyl array are used for epigenome studies, to obtain information on DNA methylation (MET). RNA sequencing (RNA-seq) and microarray are used for transcriptome studies, to obtain information on gene expression (EXP) and splice variant (SPV). Reverse phase protein array (RPPA) and liquid chromatography-mass spectrometry are used for proteome studies, to obtain information on protein expression (EXP). We implemented some standardized processes to extract data for all obtained models, including feature types, gene symbols, drug names, tissue types, cancer types, and model descriptions, thus producing 12 categories of models for 1040 drugs and 4420 genes for 144 cancer types of 30 tissues. To build an efficient research resource for pharmacogenetics, we integrated various types of information from other public resources about drugs, including chemical composition, structure, target, signal pathway, and classification, as well as information about genes, including functional description, gene expression in normal tissues, associated functions, and signaling pathways.

Finally, we designed an online web service with the interactivity of related public resources (Figure 1B). Furthermore, on the basis of our data collection, we have analyzed three cases (Figure 1C) to further illustrate the necessity of integrating the genetic models and to demonstrate the inherent value of these data. iGMDR is currently the largest resource of pharmacogenetic models in cancer, and it is freely accessible at https://igmdr.modellab.cn.

## Data collection and database content

*In vivo* models come from a large number of population studies or preclinical studies of model organisms, and most of the *in vitro* models come from the evaluation of the significant relationship between the drug and the genetic characteristics in the cancer cell line by calculation. Currently, many genetic models, including those clinically validated and experimentally validated for the evaluation of drug efficacy, have been created through *in vitro* and *in vivo* cancer pharmacogenetics studies (Figure 1A).

### *In vivo* genetic models for pharmacogenetic studies

We collected *in vivo* genetic models currently used in clinical practice from existing clinical institutes, societies, consortiums, or associations including NCCN, FDA, AACR, (http://cancerres.aacrjournals.org/), American Society of Clinical Oncology (ASCO, (https://am.asco.org/), ClinicalTrails.gov (https://clinicaltrials.gov/), and European Society for Medical Oncology (ESMO, https://www.esmo.org/). In addition, we searched the literature in PubMed for the keywords “Biomarker/cancer/tumor/drug” for nearly five years (from August 2012 to August 2017), obtaining more than 25,000 literature abstracts, and then performed the manual screening. We collated the results of pharmacogenetic studies from ~6000 literature publications, most of which came from *in vivo* studies.

### *In vitro* genetic models for pharmacogenetic studies

Most of our collection of *in vitro* genetic models came from the analysis of the results of drug sensitivity tests using cell lines. These are several large pharmacogenetic and pharmacoproteomic studies including GDSC, CTRP V2, CCLE, and MCLP. Although model production is not determined by a single drug concentration and cell line, note that the production of these models is based on cell lines with different concentrations of drug sensitivity testing, and they refer to different evaluation indexes, for example, the activity area (also called the area over concentration response curve) and IC_50_ estimation; as such, the collected models are based on their respective criteria. Although these models arise by using different criteria, subsequent analysis in many studies have revealed that these models are generally used and are statistically significant [11].

### Logic models and single models

The genetic models we collected were divided into simple models (characterized by a single genetic characteristic) and logical models (characterized by logical combinations of multiple characteristics). The logical models include the logic “and” (denoted as &), which represents the union of two genetic characteristics; logic “or” (denoted as |), which indicates that either of the two genetic characteristics can be replaced with each other; and logic “not” (denoted as ¬), which indicates that the relevant characteristic is not detected in cancer. The logical combination improves outcomes of the likelihood of predicting drug response, which helps us to understand action mechanisms of drug response [14].

### Feature types in the model

As mentioned above, different genetic characteristics are generated according to the technical background and the characteristic-related recognition methods. The types of the models are composed of 40 subclasses of 12 categories that depend on different feature events (also called genetic characteristics) (Table S1t), among which the most important 6 categories include feature events such as SNV, CNV, EXP, SV, SPV, and cell lineage (LN).

### Integration of cancer types and anticancer drugs

As the names of cancer types collected from different data sources are not uniform, it is inconvenient for the data to be normalized for analysis. Therefore, we adopted the OncoTree (http://oncotree.mskcc.org) to standardize the cancer names and related tissue types. We acquired cancer genetic models from 144 types of cancer and 30 types of cancer tissues with these processes. Names of anticancer drugs also vary according to different data sources. Therefore, we manually standardized the information on drugs and associated it to the common databases such as DrugBank [15] and PubChem [16]. In doing so, we not only normalize the drug information but also promote the interactive function with other databases. Consequently, information on 1040 drugs or drug combinations is standardized in iGMDR.

### Annotation of drugs

The target of a drug (direct action gene) and the signal pathway of drug action are important for the study of the therapeutic mechanism of the drug. The drug target information involved in the model was obtained from the Therapeutic Target Database (TTD) [17] and its source databases such as GDSC and CTRP. The signal pathway information related to drug therapy was collected from GDSC, CTRP, and CCLE. In addition, drug classification information is collected from DrugBank, and structural information on compounds (small molecule drugs) comes from PubChem.

### Annotation of genes

All of the genetic models we collected that characterize drug response involve related genes, including common oncogenes and tumor suppressor genes, as well as signaling genes involved in the pathogenesis of cancer. The basic information on these genes was collected from the MyGene application interface (API) [18]. In addition, we used the OMIM database (http://www.omim.org) to correlate the disease information with related genes, and the gene-related drug response information was accessed using PharmGKB [19]. Gene-related functions and gene-involved signaling pathways were also annotated through the Gene Ontology (GO) and Pathway databases (KEGG, Reactome, Wikipathways, *etc*.). We also collected the expression values of these genes in different normal tissues from the NCBI BioProject database to help user understand the expression backgrounds of genes in different cancer tissues.

### Comparison with existing databases

Currently, genetic models of cancer drug response are buried in individual studies, such as CCLE and GDSC (Table 1). The data within these resources come from their own pharmacogenetic studies, which are not well organized and integrated. Thus, it is difficult to effectively use the scattered information to promote the macroscale research on drug therapies and the mechanistic research on individual drug responses in cancers. The CCLE database examined the drug responses to 24 drugs in 1000 cancer cell lines, and GDSC examined the drug responses of 266 drugs in 1065 cancer cell lines. There are many differences in both the cell lines and the types of drugs tested in the various sources of the model information, as well as in the naming of cancer types and drugs. For this reason, we integrated their data and constructed our database, iGMDR, with over 154,000 predictive models of over 1000 drugs. iGMDR not only contains the model information on *in vitro* cell line experiments but also collects *in vivo* experimental models, which greatly expands the application value of relevant data in pharmacogenetic studies. When we developed iGMDR, another team also developed a database of disease related knowledge, PreMedKB [20], which included 7.94% of cancer-related precision medicine knowledge. Although PreMedKB is built with different goals from ours, some of the data we collected were from the same sources. Based on the cancer-related data collected for PreMedKB, we compared several cancer types with iGMDR (the PreMedKB website does not provide a complete list of cancers, so we cannot capture all cancer-related models at once). The results are presented in Table S2, which shows that there are far more data at the protein and gene levels in iGMDR than in PreMedKB. Moreover, a complete combination of cancer–gene–feature–drug can be called a model in iGMDR. However, the concept of the semantic network in PreMedKB can only express the relationship between two of these (*e.g.*, cancer–gene, feature–cancer, or drug–gene). Therefore, it is difficult for users to obtain the model directly, which requires manual screening within the semantic network. As a result, it is more difficult to use PreMedKB data for systematic analysis.

**Table 1:** **Comparison of existingdatabases with iGMDR**

**Database**	**No. of drugs**	**No. of cancers**	**No. of models**	**No. of feature categories**	**Feature level**	**Weblink**	**Ref.**
CCLE	15	ND	600	2	Gene/ cell lineage	https://portals.broadinstitute.org/ccle	[10]
GDSC	217	17	1648	4	Gene	https://www.cancerrxgene.org/	[11]
CTRP	202	8	132,027	4	Gene/ cell lineage	http://portals.broadinsitute.org/ctrp/	[12]
MCLP	539	17	5735	1	Protein	http://tcpaportal.org/mclp/	[13]
iGMDR	1040	144	154,146	12	Gene/ protein/ cell lineage	https://igmdr.modellab.cn	Current study

*Note*: ND, not defined.

### Data statistics

**Figure 2:**
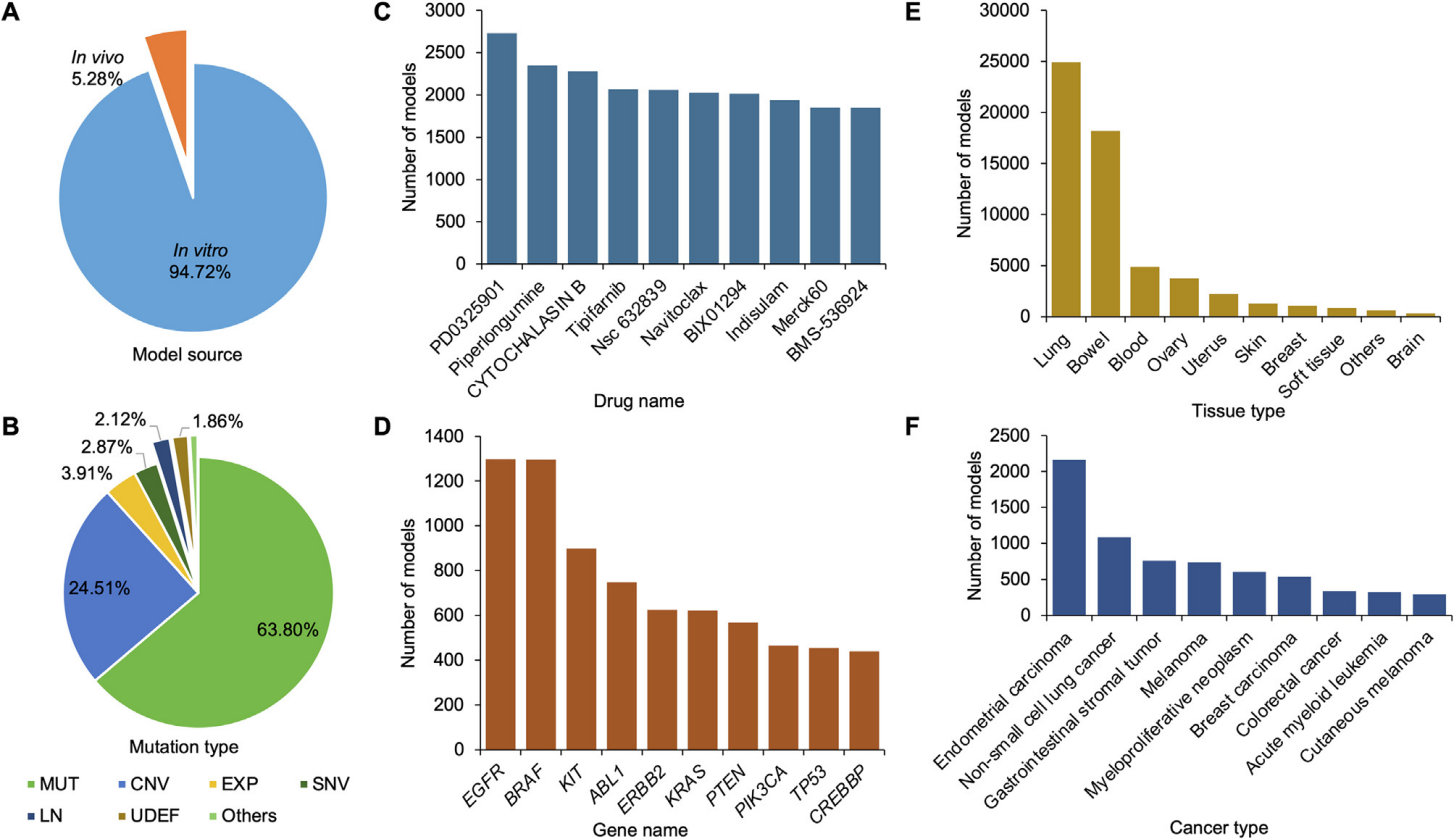
**Statistics of the models in iGMDR** **A.** The percentage of genetic models *in vivo* and *in vitro*. **B.** The percentage of mutation types that are associated with the genetic models. **C.** The top ten drugs with the largest number of associations with the genetic models. **D.** The top ten genes with the largest number of associations with the genetic models. **E.** The top ten tissues with the largest number of associations with the genetic models. **F.** The top ten cancers with the largest number of associations with the genetic models. MUT, mutation; CNV, copy number variation; EXP, expression; SNV, simple nucleotide variation; LN, cell lineage; UDEF, undefined gene status; Others (SV, structural variation; PW, pathway; SPV, splice variant; WT, wild type; PHOS, phosphorylation; MET, methylation).

The iGMDR database contains 154,146 genetic models of 144 cancer types of 30 tissues that are associated with 1040 drugs and 4420 genes. As described above, these models based on different sources were classified into *in vivo* and *in vitro* classes, and the related feature events were categorized into 12 main types. For a better understanding of the data structure of iGMDR, we conducted statistics based on the number of genetic models. As shown in Figure 2A, a majority of the models is *in vitro* (94.72%), with only 5.28% from *in vivo* systems. In this regard, more efforts are required to develop these *in vitro* models into *in vivo* models. Among the 12 main types of feature events, the top six types with the largest numbers of models are mutation (MUT; 63.80%), copy number variation (CNV; 24.51%), expression (EXP; 3.91%), single nucleotide variation (SNV; 2.87%), cell lineage (LN; 2.12%), and undefined gene status (UDEF; 1.86%), whereas other types only represent a small percentage (total 0.93%) of models (Figure 2B). This means that researchers may have overlooked many important models from other types, more attention to these feature events should be paid for developing additional genetic models.

We also collated all data to produce rankings of genes, drugs, tissues, and cancers by the number of models. The top 10 ranks with the largest numbers of models are shown in Figure 2C–F. Most of the drugs at the top of the list are small molecules (Figure 2C), because small-molecule drugs are still the mainstay of cancer therapy and are often used in cancer cell line tests. As shown in Figure 2D, the top rankings include proto-oncogenes (*EGFR*, *ERBB2*, *BRAF*, *ABL1*, *KRAS*, *KIT*, *PIK3CA*, *TP53*, *PTEN*, and *CREBBP*) and tumor-suppressor genes (*TP53*, *PTEN*, and *CREBBP*), both of which play key roles in the development and progression of most cancers. Many of these models have been used in clinical practice. For example, MUT(*EGFR*) is a predictive model for the use of afatinib, erlotinib, and pembrolizumab in non-small cell lung cancer patients. The tissues of lung, bowel, blood, and ovary have the most genetic models with cancer therapies (Figure 2E). This observation is consistent with the frequency of mutations in associated cancers based on the statistics of the COSMIC database (https://cancer.sanger.ac.uk/). Interestingly, of the cancers with the highest number of genetic models, endometrial carcinoma far outweighs non-small cell lung cancer, whereas acute myeloid leukemia represents slightly less than melanoma (Figure 2F). This observation does not match the results in the tissues, and it may be due to differences in the number of cancers present in different tissues, thus diluting the number of models for a specific type of cancer.

## Database implementation

### Webserver and API construction

iGMDR was built in the Apache HTTP server, and all model data were stored in a MySQL database. PHP was mainly used for backstage and front-end interaction. HTML and JavaScript were used for front-end rendering, and bootstrap and d3.js were used to efficiently improve data presentation and visualization. The webserver code is available on the GitHub repository (https://github.com/ModelLAB-ZJU/iGMDR) based on the GPLv3 license. User manuals can be queried on webserver's documentation page.

To facilitate data utilization, we have released the model data not only through an online web server but also through an API. The API defined by the swagger platform provides simple-to-use web services to query/retrieve model data, ensuring that users can interact with other tools to use the drug response model information we collected. The API is an architecture of representational state transfer (REST), meaning that all database resources can be located using URLs, and the operations are described using HTTP verbs (get, post). The iGMDR API uses PHP to perform parsing without setting any user password and can be used publicly. “Post” and “get” requests are used for all queries, returning information in JSON format. In addition, we also provide a dump of the SQL database for download (see the “iGMDR access” column on Figure 1A).

iGMDR's web application accepts any feedback about database content and data presentation, and users can operate in the GitHub platform or communicate using email directly. In addition, we believe that with the development of pharmacogenetic research, the data collected by iGMDR currently are still limited, users are welcome to recommend new data sources. The increase in the amount of data will further boost its value.

### Usage

**Supplementary Figure S1:**
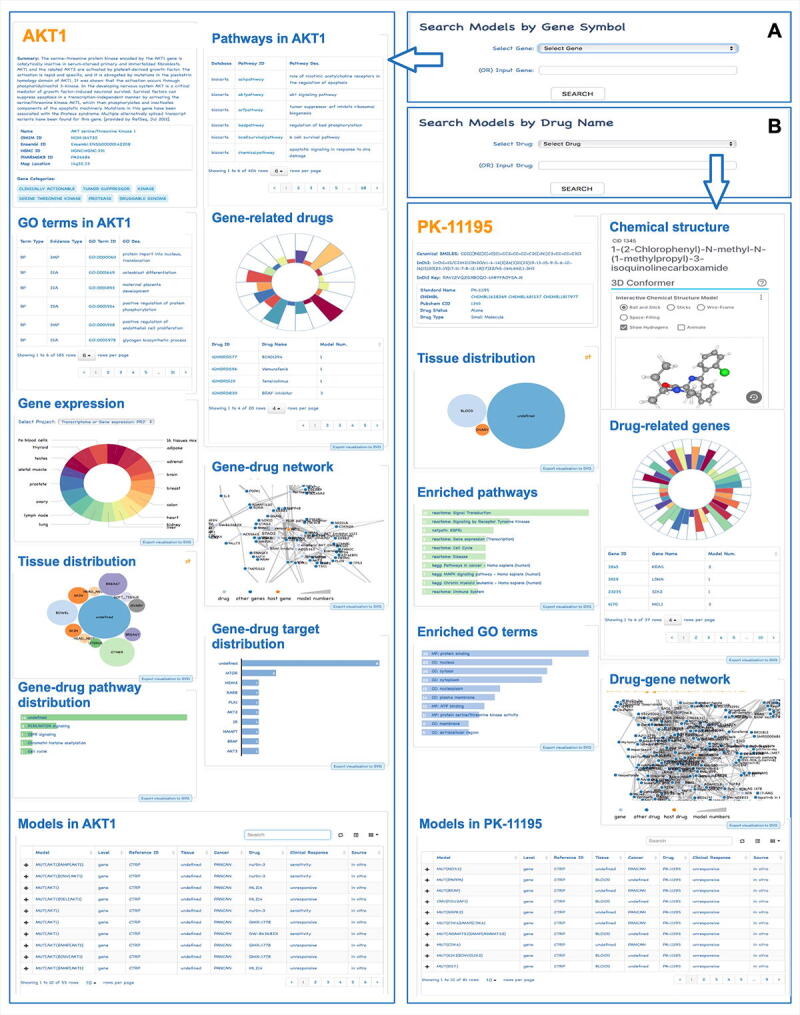
**Searching models in iGMDR. A.** Model and profile searching by inputting a gene symbol. **B.** Model and profile searching by inputting a drug name.

Intuitively, pharmacogenetics researchers are often interested in genes involved in the model and the related drugs. We thus designed the relevant search interface. For these searches, we offer two methods: a drop-down menu of search items or manually entering keywords (Figure S1). The drop-down menu can effectively prompt the user to enter whether the information is present or not, which is convenient for the user to inquire directly. Here, we used *AKT1* (gene) and PK-11195 (drug), respectively, as examples to illustrate the use of iGMDR. By searching for drugs and genes, users will be directed to a profile page for the gene *AKT1* (Figure S1) or the drug PK-11195 (Figure S1), and the feedback data will be displayed through different visualizations (Figure S1). The drug exhibitions include the attribute information of PK-11195, associated response models in PK-11195, tissue origin distribution of these models, function and signaling pathway enrichment of the model-related genes. Specifically, the attribute information of PK-11195 covers chemical composition, structure, classification, target, signaling pathways, and associated external database ID. Moreover, drug–gene relationship network between the model-related genes and the drugs related to these genes are displayed for users, and the strength of the relationships illustrated by the line weight in the network is calculated according to the number of the associated models. The gene profile describes the basic information on *AKT1*, including summarized gene function, gene categories, and associated external database ID. The *AKT1*-related response models, *AKT1*-involved function and signaling pathways, expression distributions of *AKT1* gene in normal tissue, tissue origin distributions for *AKT1*-related models, *AKT1*-related anticancer drugs, and enrichment of these drug-related targets and signaling pathways are also available to users. The drug–gene network is constructed based on the number of associated models to find the gene-related drugs and genes related to these drugs. Similarly, the strength of the relationships is illustrated by the line weight. Note that these results profiled for the gene and drug are based on the gene/drug-associated models, which come from different types of cancer, different references, and different sources. For convenience, we designed a smart table that provides filtering to view related models based on these options (Figure S1).

**Supplementary Figure S2:**
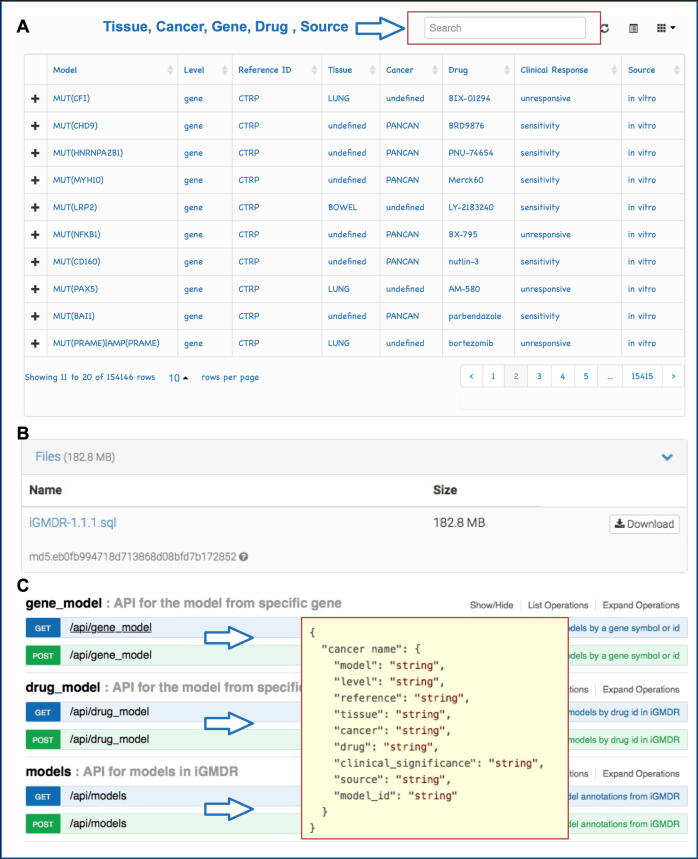
**Exploring models in iGMDR. A.** Model exploration by using a search box with different keywords. **B.** Downloading all tables of the database from zenodo.org for exploring the models. **C.** API calls return a json object for exploring the models.

To unlock the value of big data, users often need to systematically analyze all model data. Therefore, the browse page is essential for users to explore iGMDR, where specific information can be viewed through a smart table (as mentioned above) (Figure S2). In addition, we have also provided classified browsing through various data types, including data sets, tissue types, cancer types, specific drugs, and specific genes (see the bottom navigation bar at https://igmdr.modellab.cn). For users to implement local profiles, raw database tables can be downloaded from zenodo.org (Figure S2). Furthermore, the API provides object data for other tools to profile or visit the genetic models in different channels, *i.e.*, by gene symbol or drug name (Figure S2).

## Discussion

### Design of new panels for cancer care

**Figure 3:**
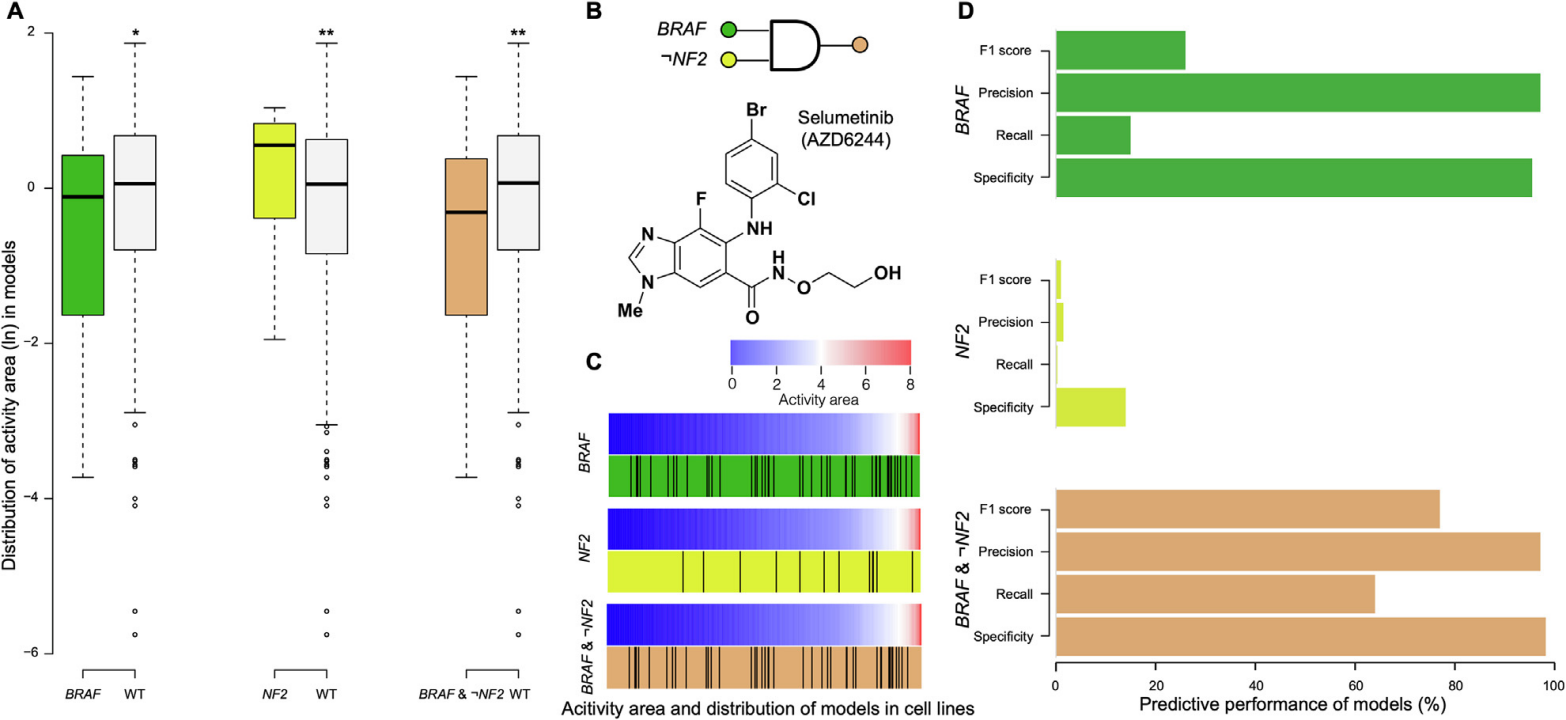
**The logical combination of*****BRAF*****and*****NF2*****improves the predictive efficiency for anticancer drug AZD6244** **A.** The distribution of activity area in anticancer drug response of cell lines with different model characteristics for single characteristic MUT(*BRAF*/*NF2*) or logical combination MUT(*BRAF*) & ¬MUT(*NF2*). **B.** The pattern of combinations and the molecular composition of AZD6244. **C.** Distribution of the three models with respect to the drug sensitivity of different cell lines. Activity area (the area over the dose–response curve) are color coded from blue (resistance) to red (sensitivity). The vertical lines represent the cell lines with the model characteristics. **D.** The evaluations of the prediction via different models., **, *P* < 0.001; *, *P* < 0.01 (HyperG test). See Table S3 for the detailed data analysis.

Clinical genome sequencing is being increasingly applied to clinical practice, and it offers promising prospects for personalized cancer therapy [21]. It is known that anticancer drugs respond differentially in different patients because of the heterogeneity of the same tumor in different individuals [22]. How to predict the therapeutic outcome of anticancer drugs effectively according to the clinical sequencing analysis of patients is an important aspect of personalized therapy (see the “Therapeutic response models” and “Novel therapy strategies” columns in Figure 1C). Here, we can use the predictive models we have collected to design new clinical sequencing panels that predict the efficacy of anticancer drugs. Conventional panels are almost always based on targeting genes related to anticancer drugs or oncogenes, and these panels cover only a small number of cancer patients. Similar results have been obtained in some studies [23]. For example, in the mitogen-activated protein (MAP) kinase signaling pathway, proliferation and survival may be activated via downstream gene mutations. BRAF is a central mediator in the MAP kinase signaling cascade and exerts effects predominantly through phosphorylation and activation of MEK, which has been implicated in the pathogenesis of several cancers, including melanoma, non-small cell lung cancer, colorectal cancer, papillary thyroid cancer, and ovarian cancer [24]. While AZD6244 (selumetinib) is a high-potency MEK inhibitor, resistance to therapy and tumor progression occurs in some patients with *BRAF* mutations [25]. Interestingly, this finding is not consistent with our observations, which further suggests that the judgment based on a single model is biased and only covers a subset of patients. In virtually every AZD6244 case we have observed through comparing all cell lines, we found that cell lines with *BRAF* mutations [MUT(*BRAF*) to AZD6244 model] had higher drug sensitivity than wild-type cell lines (HyperG test, *P* < 0.01), while cell lines with *NF2* mutations [MUT(*NF2*) to AZD6244 model] showed significantly lower drug sensitivity (*P* < 0.001) (Figure 3A). When we combined the two independent models, we found that the predicted drug sensitivity of more cell lines was consistent with the actual test results (Figure 3A–C). The predicted results of the combined model [MUT(*BRAF*) & ¬MUT(*NF2*) to AZD6244 model] increased the sensitivity of the single model by nearly 50%, and the specificity reached 98.2% (Figure 3D).

The combination of the two features can effectively enhance the predictive effect of drug response, and this finding is confirmed in our integrated data of the logical model in iGMDR. In addition, the model of drug response in the genome was also demonstrated at the proteomic level (for example, EXP(*EGFR*) is sensitive to AZD6244 at both protein and gene levels for breast cancer). Therefore, we can not only use data sets to discover new and more efficient drug response models of anticancer therapy, but also design new panels of cancer clinical sequencing based on model integration.

### Linking drug targets and pathway activation to effective therapy

We have collected drug targets and targeted signaling pathways of model-related anticancer drugs in iGMDR, which can extend the use of model data (see the “Mechanism of drug action” column on Figure 1C). It is well known that the use of combination drugs is of enormous value for cancer therapies, and in some cases, it effectively improves the survival time of cancer patients and controls the development of tumors. There are many successful drug combinations that have been used in clinical practice. Determining how to design new combination drugs to improve the efficacy of cancer therapy and enhance modeling to reflect the sensitivity of anticancer drugs will broaden our design conceptualization.

**Figure 4:**
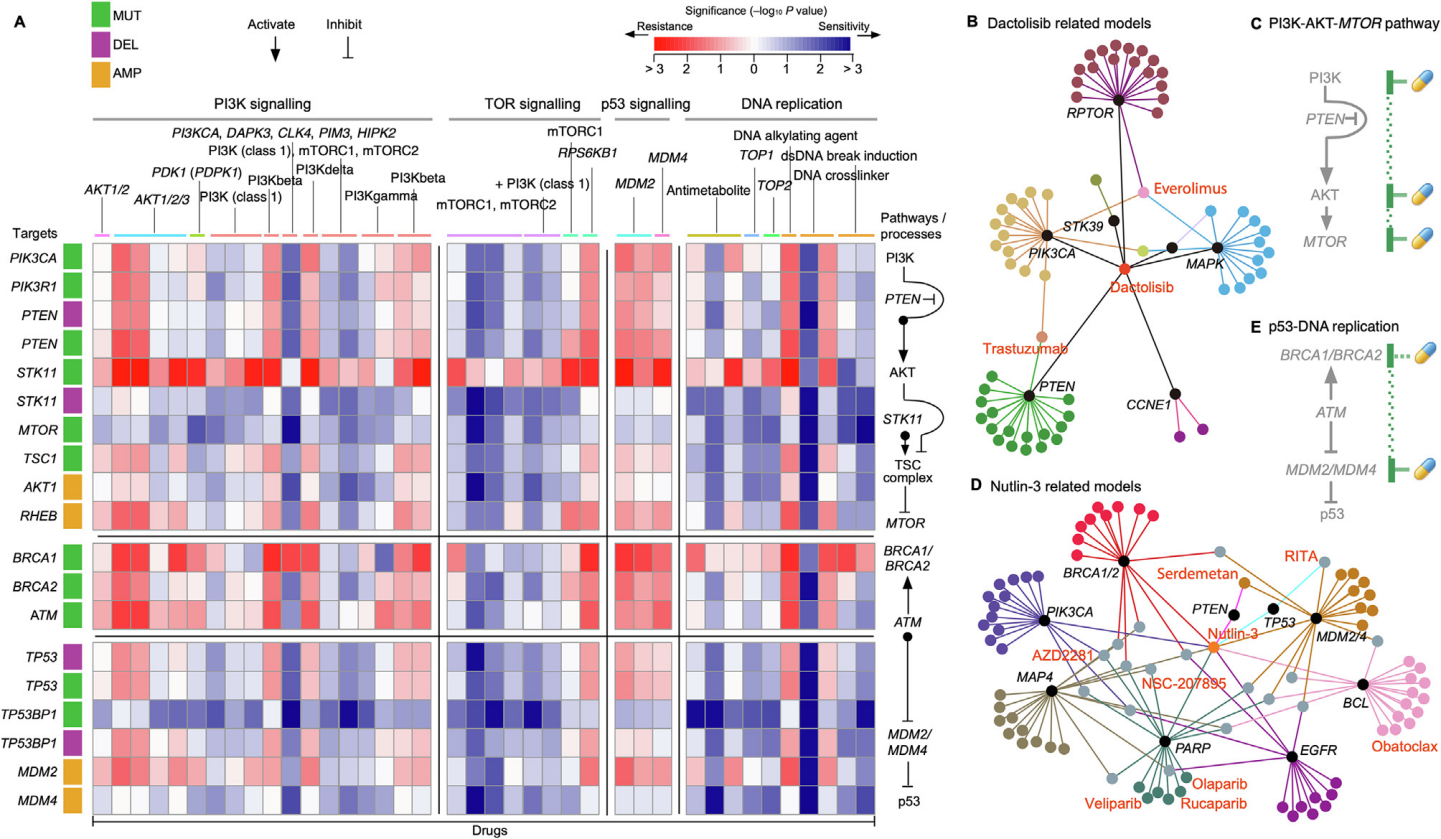
**Drug target and pathway information to increase therapeutic options** **A.** Pathway-centric overview of the collected pharmacogenetic models. Major oncogenic pathways include PI3K-AKT pathway, TOR signaling, p53 signaling, and DNA replication. The model-related genes (rows) encoding components of the oncogenic pathways are also shown schematically in the pathways (column). The cells are color-coded according to the corresponding −log_10_*P* values (for the analysis data, see Table S3). **B.** Dactolisib-related model genes that interact with other anticancer drugs. **C.** Combination strategies targeting the carcinogenic PI3K-AKT-*MTOR* pathway. **D.** Nutlin-3-related model genes that interact with other anticancer drugs. Networks were constructed through the “drug–gene network” in iGMDR. **E.** Combination strategies targeting the carcinogenic p53–DNA replication pathway.

Currently, many anticancer drugs are used and tested for important cancer-related signaling genes. For example, the phosphatidylinositol 3′-kinase (PI3K)-AKT-mechanistic target of rapamycin (*MTOR*) signaling pathway (PI3K-AKT-*MTOR*) regulates fundamental cellular functions such as transcription and translation, cell growth and proliferation, as well as regulation of apoptosis and autophagy. The PI3K-AKT-*MTOR* pathway may be activated by the binding of growth factors to their corresponding receptor tyrosine kinases (RTKs) or by activating mutations in *PIK3CA/PIK3R1*, *AKT1*, *TSC1*, and *MTOR* complex, or inhibited by phosphatase and tensin homolog (*PTEN*) [26], [27], [28], [29]. Dactolisib acting as a dual inhibitor inhibits *MTOR* and PI3K and is being investigated as a possible anticancer therapy [30], [31]. Everolimus, an approved inhibitor of mTOR, was used in the treatment of various tumors and can lead to a hyperactivation of AKT via inhibition of the mTOR complex 1 (mTORC1) negative feedback loop [32]. As predicted by the model, however, the variations in these genes will affect the potency of anticancer drugs and even generate resistance (Figure 4A). This is true for many cases where a single anticancer therapy often leads to drug resistance when a gene is mutated. Inspiringly, based on the network of drug-related models, we find that different anticancer drugs act on host drug-related genes in the network (Figure 4B). The combination of these anticancer drugs may allow the design of new strategies to increase drug sensitivity and therapeutic efficacy (Figure 4C). Studies have confirmed that mTOR inhibitor (everolimus) combined with trastuzumab reversed trastuzumab resistance via the hyperactivated PI3K-AKT-*MTOR* pathway due to *PTEN* deficiency in patients with HER2-positive advanced breast cancer [33]. Trastuzumab was approved for clinical use in HER2-positive breast cancer and works by binding to the RTK (Erb-b2 receptor) and slowing down cell replication [34]. Moreover, the combination of everolimus and dactolisib demonstrated synergy in a clinical trial as well [35].

Nutlin-3 is a commonly used mouse doubleminute 2 homolog (MDM2) antagonist that can penetrate cell membranes. It is highly selective and inhibits the interaction of MDM2-p53, thereby activating the p53 pathway, inducing apoptosis, and playing an anti-tumor role. More relevant anticancer regimens were observed (Figure 4D) through its associated model network. These drugs act mainly on DNA replication [36] (veliparib, olaparib, talazoparib, rucaparib, and sorafenib) and apoptosis signaling [37] (obatoclax, serdemetan, NSC-207895). Veliparib, olaparib, talazoparib, and rucaparib are poly real (ADP-ribose) polymerase (PARP) inhibitors. Blocking PARP in cancer cells may help prevent cancer cells from repairing their damaged DNA, causing them to die. Combined treatment of Nutlin-3 with PARP inhibitors increased cell cycle arrest and apoptosis, which was marked for preclinical trials [38]. Additionally, the combination treatment of serdemetan and obatoclax completely eliminates, or in some cases completely prevents, the onset of cancer *in vivo* (Figure 4D and E).

In conclusion, the anticancer drug models we collected could identify different combinations of anticancer drugs associated with the same cancer, and we used drug-related gene–drug networks to discover the most effective pharmacological strategies for cancer therapies.

### Tissue specificity of drug sensitivity

**Figure 5:**
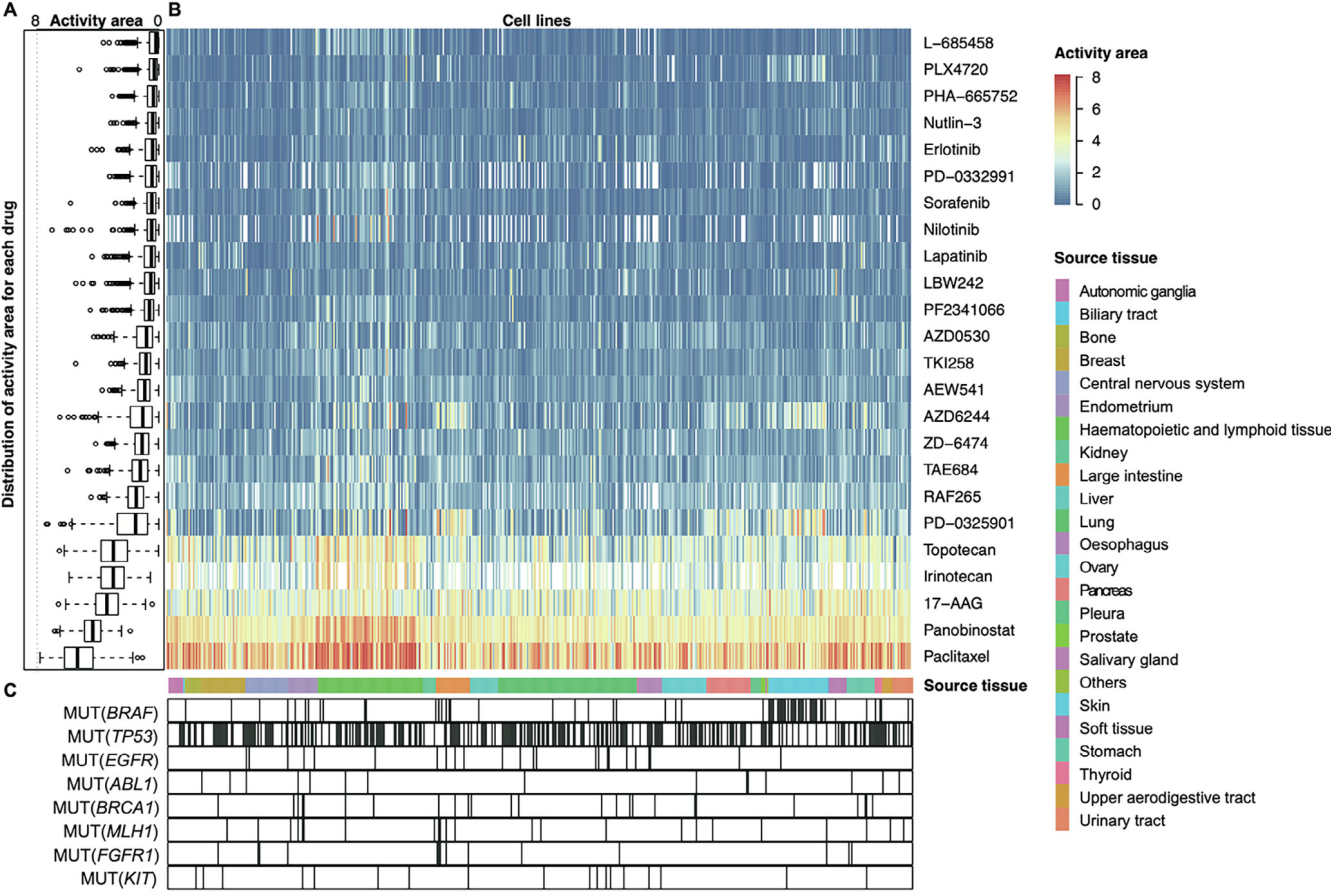
**Genetic dependencies targeted by anticancer drugs in different tissues** Activity area is used for sensitivity assessment of anticancer drugs in each cell line. **A.** Boxplot indicating the distribution of activity area for each drug (rows). **B.** Heatmap indicating the sensitivity to the anticancer drugs of different cell lines (columns), ranging from 0 (resistance) to 8 (sensitivity). The data values missing in the heatmap will be filled by the *k*-nearest neighbor method. **C.** The distribution of activity area analyzed by integrating the characteristics of the model exclusions in different cell lines and associated tissues. The vertical line indicates the presence of corresponding characteristic in the cell line. See also Table S4 for more details.

The response to the same drug by tumors of different tissues can be very different because of the tissue specificity of different cancers [39], [40]. The current tissue specificity of cancer pathogenesis has been analyzed from a variety of perspectives but has not been analyzed at the level of genetic models of anticancer drugs. Here, with the help of the collected models, we have observed enormous differences in the genetic models and drug sensitivities from different tissue types (Figure 5). Overall, the sensitivity of different drugs to all cancers is very different (Figure 5A). Some of the tested drugs are very specific and only effective against individual cancers, while some drugs cover almost all cancer cells (Figure 5B). For example, PD-0325901 is more effective against cancers derived from large intestine and skin, and panobinostat is effective against almost all cancers of tissue origin (Figure 5B). It can also be seen from the figure that cancers from each tissue origin exhibit different response patterns to various drugs. For example, haematopoietic and lymphoid tissue had stronger drug sensitivity compared with other tissues as a whole. Skin tissue was more sensitive than cancer derived from other tissue origins with respect to AZD6244 and PD-0325901 (Figure 5B). Interestingly, we found that the genetic characteristics associated with the model had different enrichment patterns in cell lines from different tissue origins. For example, MUT(*BRAF*) occurs mainly in skin tissue compared to the ubiquity of MUT(*TP53*). More interestingly, we found a correlation between this enrichment pattern and the drug sensitivity response. For example, large intestine cell lines that harbored MUT(*MLH1*) and MUT(*FGFR1*) are more sensitive to the anticancer drug paclitaxel. The breast cell lines that gained MUT(*BRAF*) had lower sensitivity to most drug treatments (Figure 5C).

The model-related genes and drug rankings of different cancers and tissues are listed in Table S5 and Table S6, respectively. It can also be seen from the schedule that there are significant differences in the priority of cancer-sensitive drugs and indication genes from different tissue origins. This is a significant resource for tissue-specific studies of tumorigenesis. Finally, with the model data, we will be able to analyze tissue-specific treatment regimens and key genes involved in the development of specific cancers.

### Summary and future directions

Our goal is to integrate more comprehensive data to discover new knowledge and explore promising strategies for cancer therapies. iGMDR is the first complete data resource to provide predictive models for anticancer drugs, and it is by far the largest resource. It provides not only a normalized exhibition of model data but also an investigation of the response models of anticancer therapies for individual genes and individual drugs. The interactive and visual presentation of data directly presents the macroscale and microscale results of drug response.

The models that we collected included both those that were related to drug sensitivity and resistance and those that were unresponsive because we believe that this would be valuable information for clinical practice or research. In addition, due to the different sources of all models, the reliability varies greatly, and it is difficult to reflect its importance with unified indicators. Different models of the same gene (with different features) may have different outcomes for the same drug intervention. Therefore, we use the network to analyze the relationship between genes and drugs and rely on the number of models to judge the importance of the relationship between drugs and genes. The importance of a single model is usually judged to be more reliable *in vivo* than *in vitro*. At the same time, mutation-level model, such as SNV(*ABL1* V299L), is more reliable than the gene-level model, such as MUT(*ABL1*).

With the further development of pharmacogenetic studies and high-throughput technologies, relevant therapeutic response models for anticancer drugs will continue to be updated, and we will continue to focus on data replacement to improve data breadth, quality, and objectivity. Some of the models consider the efficacy of the combination and continuous use of different drugs over a period of time, which are priorities. In our previous work on model matching to patients (unpublished data), we designed a logical strategy similar to the aforementioned logic model to process relevant data. In addition, we will provide a new angle to analyze data by associating it with new databases. For example, the Connectivity Map (CMAP, https://clue.io) [41] and Library of Integrated Network-Based Cellular Signatures (LINCS, http://www.lincsproject.org) [42] datasets, which provide information about drug perturbations, will allow users to combine these datasets with the response models of anticancer therapies to better understand the mechanisms of action of drugs.

Finally, iGMDR will focus on integrating data from pharmacogenetic studies to increase the value of the data as much as possible, to facilitate clinical studies and practices.

## Data availability

iGMDR is freely accessible at https://igmdr.modellab.cn and is intended for academic purposes only.

## CRediT author statement

**Xiang Chen:** Conceptualization, Methodology, Investigation, Software, Visualization, Writing - original draft, Project administration. **Yi Guo:** Data curation, Formal analysis, Investigation, Writing - review & editing. **Xin Chen:** Funding acquisition, Resources, Supervision, Writing - review & editing. All authors read and approved the final manuscript.

## Competing interests

The authors have declared no competing interests.
