# Supplementary material for: iGMDR: Integrated Pharmacogenetic Resource Guide to Cancer Therapy and Research
Source: Genomics Proteomics Bioinformatics. 2020 Sep 8;18(2):150–60. doi: 10.1016/j.gpb.2019.11.011 (PMC7646137; doi:10.1016/j.gpb.2019.11.011)
Supplement: Supplementary Table S1 [file mmc1.docx]

**Table S1 The types of feature events (mutations) in iGMDR models**

| **Main type** | **Subtype** | **Subtype description** | **Examples** |
| --- | --- | --- | --- |
| CNV | AMP | Amplification | Amplification; CNV-H |
| MUT | BIA-IA | Biallelic inactivation | Biallelic inactivation |
| CNV | CNV | Copy number variation | CNV |
| CNV | DEL | Deletion | Deletion; copy-neutral loss of heterozygosity; CNV-L |
| CNV | DEL-MUT | Deleterious mutation | Deleterious mutation |
| CNV | DEL-PLM | Deletion polymorphism | Deletion polymorphism |
| MUT | D-MUT | Domain mutation | Exon nucleus domain mutation; tyrosine kinase domain mutations |
| EXP | EXP | Expression mutation | Expression; serum levels |
| MUT | FL | Function loss | Any function loss |
| MUT | FS-MUT | Frameshift mutation | Frameshift mutation |
| SV | FUS | Fusion | Fusion; translocation; rearrangement |
| MUT | GT | Genotype | OncoGeno; homozygosity |
| MET | HM | Hypermethylation | Promoter hypermethylation |
| SNV | IF-DEL | Inframe deletion | Inframe deletion |
| SNV | IF-INT | Inframe insertion | Inframe insertion |
| SNV | INDEL | Insertion/deletion | Insertion / deletion |
| SNV | INT | Insertion | Insertion; InsT |
| SPV | ISOF | Isoform | Isoform; alternative transcript; isoform expression |
| SNV | ITD | Internal tandem duplication | Internal tandem duplication; tandem repeat |
| LN | LN | Lineage | Lineage |
| MET | MET | Methylation | Methylation |
| SV | MISL | Mislocation | Mislocation |
| MUT | MUT | Mutation | Activating mutation missense |
| EXP | NC-EXP | Nuclear expression | Nuclear expression |
| EXP | OEXP | Overexpression | Overexpression |
| MUT | ONCO-MUT | Oncogenic mutation | Oncogenic mutation |
| PHOS | PHOS | Phosphorylation | Phosphorylation |
| SNV | PLM | Polymorphism | Polymorphism |
| PW | PWD | Pathway down-regulation | PWD |
| PW | PWU | Pathway up-regulation | PWU |
| MUT | SKP-MUT | Skipping mutation | Skipping mutation |
| LN | SLN | Sublineage | Sublineage |
| SNV | SNV | Single nucleotide variation | SNV |
| SPV | SPAV | Splice acceptor variant | Splice acceptor variant |
| SPV | SPDV | Splice donor variant | Splice donor variant |
| SPV | SPV | Splice variant | Splice variant; splice site insertion |
| SNV | TC-MUT | Truncation mutation | Truncation mutation |
| UDEF | UDEF | Undefined gene status | Gene |
| EXP | UEXP | Underexpression | Underexpression |
| WT | WT | Wild type | Wild type |

*Note*: MUT, mutation; CNV, copy number variation; CNV-H, CNV high (copy number higher than normal); CNV-L, CNV low (copy number lower than normal); EXP, expression; SNV, single nucleotide variation; LN, cell lineage; UDEF, undefined gene status; SV, structural variation; PW, pathway; SPV, splice variant; WT, wild type; PHOS, phosphorylation; MET, methylation; OncoGeno, oncogenic genotype; InsT, insertion.
