# Supplementary material for: iGMDR: Integrated Pharmacogenetic Resource Guide to Cancer Therapy and Research
Source: Genomics Proteomics Bioinformatics. 2020 Sep 8;18(2):150–60. doi: 10.1016/j.gpb.2019.11.011 (PMC7646137; doi:10.1016/j.gpb.2019.11.011)
Supplement: Supplementary Table S2 [file mmc2.docx]

**Table S2 Comparison of PreMedKB with iGMDR**

| **Cancer type** | **No. of models** | |  | **No. of drugs** | |  | **No. of genes** | |  | **Feature level** | |
| --- | --- | --- | --- | --- | --- | --- | --- | --- | --- | --- | --- |
|  | **iGMDR** | **PreMedKB** |  | **iGMDR** | **PreMedKB** |  | **iGMDR** | **PreMedKB** |  | **iGMDR** | **PreMedKB** |
| Non-small cell lung cancer | 1087 | 931 |  | 88 | 30 |  | 44 | 10 |  | Gene / protein | Gene |
| Acute myeloid leukemia | 324 | 0 |  | 206 | 0 |  | 37 | 0 |  | Gene | - |
| Colorectal cancer | 339 | 70 |  | 89 | 2 |  | 51 | 2 |  | Gene / protein | Gene |
